# Luo Tong Formula Alleviates Diabetic Retinopathy in Rats Through Micro-200b Target

**DOI:** 10.3389/fphar.2020.551766

**Published:** 2020-10-30

**Authors:** Bing Pang, Qing Ni, Sha Di, Li-juan Du, Ya-li Qin, Qing-wei Li, Min Li, Xiao-lin Tong

**Affiliations:** ^1^ Department of Endocrinology, Guang’ Anmen Hospital of China Academy of Chinese Medical Sciences, Beijing, China; ^2^ Zhongshan Ophthalmic Center, Sun Yat-sen University, Guangzhou, China; ^3^ Molecular Biology Laboratory, Guang’ Anmen Hospital, China Academy of Chinese Medical Sciences, Beijing, China

**Keywords:** diabetic retinopathy, luo tong formula, prevention, microrna-200b, vascular endothelial growth factor/pigment epithelium-derived factor ratio

## Abstract

**Aim:** Diabetic retinopathy (DR) is a serious complication of diabetes (DM). Luo Tong formula (LTF) exerts protective effects against DR in rats, but its underlying mechanism remains unknown. **Methods**: Sprague-Dawley rats injected with streptozotocin (STZ) were used as an experimental diabetes model. LTF or calcium dobesilate (CaD) was administered to diabetic rats via gastric gavage. After the 12 weeks of treatment, blood and tissue samples were collected to determine serum glucose and retinal structure. Blood samples were collected for blood glucose and hemorheology analysis. Gene or protein expression levels were evaluated by immunohistochemistry, western blotting and/or quantitative real-time polymerase chain reaction (PCR). **Results:** DM rats exhibits significantly increased blood retinal-barrier (BRB) breakdown and VEGF/VEGFR expression in the retina, and decreased miR-200b and tight junction ZO-1/Occludin/ Claudin-5 genes expression, as well as Ang-1/Tie-2 expressions in the retina compared to normal control group. LTF treatment significantly moderated histological abnormalities in diabetic rats, independent of blood glucose level; improved some hemorrheological parameters; decreased the expressions of VEGF/VEGFR and BRB breakdown, significantly increased PEDF and tight junction proteins ZO-1/Occludin, as well as increased retinal miR-200b expression compared to non-treatment diabetic rats. Moreover, LTF prevented the reduction in Ang-1/Tie-2 expression. **Conclusions:** LTF treatment ameliorated DR through its repair vascular and attenuate vascular leakage. A mechanism involving *miR-200b* may contribute to benefit effects.

## Introduction

The rapidly increasing incidence of diabetes mellitus (DM) is becoming a major public health issue (Wang et al., 2017). Diabetic retinopathy (DR) is one of the most common and serious complications of DM, and remains the leading cause of blindness in working-age adults; DR-induced blindness has increased by 27% worldwide, and visual impairment related to DR has increased by 64% in the past 10 years (Leasher et al., 2016). Vision-threatening retinopathy is irreversible, dramatically affecting the quality of life of patients. Therefore, early prevention and treatment are necessary, and more and more early preventive measures have been paid attention (Valencia and Florez, 2017). Chinese herbal medicine has recently been shown to be effective for the prevention and treatment of DR (Pang et al., 2020b). Luo Tong formula (LTF) originated from Di Dang Decoction (*Synopsis Golden Chamber*), we previously reported that LTF prevented DR in rats. Moreover, we found that it protected endothelial cells by ameliorating inflammation and inhibiting cell apoptosis (Pang et al., 2020a; Zhou et al., 2000). However, the upstream mechanism involved remains unclear.

MicroRNAs are small noncoding RNAs on average only 22 nucleotides, which are thought to work with their target genes and regulate DR (Zhang et al., 2017). The miR-200 family, including miR-200b, is a cluster of miRNAs (Belgardt et al., 2015), suppression of miR-200b upregulates vascular endothelial growth factor (VEGF) expression in the diabetic rat retina and increases glucose-induced permeability and angiogenesis in human umbilical vein endothelial cells (HUVECs), which are highly associated with the pathogenesis in DR (McArthur et al., 2011; Ruiz et al., 2015; Li et al., 2017). Pigment epithelium-derived factor (PEDF), a potent inhibitor of angiogenesis, has been found to be associated with miR-200b and involved in the pathogenesis of DR (Zheng et al., 2009; Li et al., 2017). Hyperglycemia instigates oxidative stress, which silences miR-200b, then increase VEGF and inhibit PEDF expression, further aggravate the blood-retinal barrier (BRB) breakdown and vascular leakage, which is the key of retinopathy development (Cheung et al., 2010; McArthur et al., 2011; Kowluru et al., 2015; Ruiz et al., 2015; Li et al., 2017). Thus, targeting the expression of miR-200b may be a novel therapeutic approach for DR. In this study, we initially explored the effects of LTF on inhibiting the development of DR in diabetic rats, then determined the mechanisms underlying the effects of LTF on alleviating vascular leakage through miR-200b targets.

## Materials and Methods

The study was approved by the Ethics Committee of the Laboratory Animal Management of Guang’anmen Hospital of China, Academy of Chinese Medical Sciences (IACUC-GAMH-2020-001), and all efforts were made to minimize animal suffering. All study protocols and animal handling were performed according to the guidelines for animal use published by the Association for Research in Vision and Ophthalmology.

### Standardization of Luo Tong Formula

LTF, originated from Di Dang Decoction (*Synopsis Golden Chamber*), is a Chinese herbal formula comprising *Astragalus* mongholicus Bunge, Salvia miltiorrhiza Bunge, Panax notoginseng (Burkill) F. H. Chen, *Terminalia* chebula Retz., and Rheum officinale Baill. at a ratio of 15:10:3:2:1. Crude drugs of LTF were provided by Kangmei Pharmaceutical Co., Ltd. after standard quality measurement, and LTF was decocted in the Pharmacy Department of Guang’ anmen Hospital, China Academy of Chinese Medical Sciences under standard procedures.

### Animal Model and Treatment

Male 6-week-old Sprague-Dawley rats were purchased from Vital River Laboratory Animal Technology Co., Ltd. (Beijing, China) and acclimated for 1 week. Animals were housed in cages with a 12-h light/12-h dark cycle at 22°C. The animals had free access to food and water. Diabetes was induced by an intraperitoneal injection of streptozotocin (STZ; 65 mg/kg, in 10 mM citrate buffer, pH 4.5) in 8-week-old male SD rats. Age-matched control rats were injected with an equal volume of vehicle. Forty-eight hours after STZ injection, the blood glucose level was measured from the tail vein. Rats with a blood glucose level higher than 13.9 mmol/L (250 mg/dl) were considered to be diabetic (Leal et al., 2010). The rats were randomly allocated into four groups: 1) normal control rats treated with distilled water, 2) Diabetic rats treated with distilled water; 3) Diabetic rats treated with LTF, and 4) Diabetic rats treated with calcium dobesilate (CaD). The effective dosage of LTF in humans is approximately 1.55 g/kg/d. Rats require approximately 6.25 times the amount of a drug as patients. Thus, LTF at a dosage of 9.69 g/kg/d was administered to the rats in the LTF group; the dosage of CaD in rats is approximately 104 mg/kg/d based on the daily doses commonly prescribed in humans (Leal et al., 2010); the diabetic rats were given distilled water. LTF, calcium dobesilate (CaD) or distilled water was administered to diabetic rats via gastric gavage. After 12 weeks of treatment, the rats were anaesthetized, and the blood sample and eyeballs were collected.

### Examination of Blood Glucose and Hemorheology

Glucose measurements were taken from the tail vein twice a month and measured using OneTouch Ultra Blood Glucose Meter and test strips (Shen Zhen, China). At 12 weeks, all rats were anesthetized and euthanized, and blood samples were collected. Blood serum glucose was measured using an automatic biochemical analyzer (OLYMPUS-640; Tokyo, Japan). Hemorrheological parameters were determined with a Contraves Low-Shear 30 Sinus Rheometer (Switzerland). Fibrinogen was determined by Biuret tests.

### Retinal Histological Analysis

The right eyeballs were removed and fixed in 4% paraformaldehyde for 48 h at 4°C, and embedded in paraffin. The paraffin sections were deparaffinized and dehydrated in alcohol, then the sections (5 μm) were stained with hematoxylin and eosin (H&E). Digitized images of the retina were captured at 400× magnification by a light microscope (OLYMPUS BH-2, Japan). The results from four sections per eye were averaged. The total retinal thickness was measured at 400× magnification; two measurements were taken for each section at the two reference lines, which were 1 mm away from the optic nerve on both the superior and inferior sides. The number of the retinal ganglion cells (RGC) was measured at 400× magnification (Zhang et al., 2018). The thickness of the retinas was measured from the inner limiting membrane to the tips of the photoreceptor outer segments. All the cell nuclei within a fixed 25-mm column and centered with the 1-mm reference lines were counted (Zhang et al., 2018).

The left eyeballs were removed and fixed in 4% paraformaldehyde for 48 h at 4°C, then the retinal tissues were isolated. Samples were subsequently placed into pepsin fluid for 1 h at 37°C and trypsin fluid for 3 h at 37°C, then stained with periodic acid-Schiff (PAS) and examined under a light microscope (magnification, × 400) to calculate the number of acellular capillaries. The number of endothelial cells and pericytes were also counted, and the ratio was calculated. The images were taken by two blinded observers.

### Measurement of Evans Blue Leakage

After 12 weeks of treatment, BRB breakdown in rats was analyzed as described previously, with some modifications (Chen et al., 2017). Under anesthesia, the rats were intravenously injected with Evans blue solution (EB; 45 mg/kg) and kept on a warm pad for 120 min. The thoracic cavity of rats was then opened, and needles were inserted from the left ventricle to aortic arch. Rats were perfused with citric acid buffer at 37°C until the liver appeared white. Then, the eyeballs were removed, and the retinas were dissected immediately. The retinas were dried and weighed; dry weight was used to standardize the quantification of EB leakage. One hundred 20 mL of formamide was mixed into the retinas, and the mixtures were incubated. Finally, the mixture was centrifuged at 15,000 rpm for 45 min, and the supernatants were placed into Eppendorf tubes. The absorbance of the EB solution was measured at 620 and 740 nm, and the retina-related net absorbance value was calculated. The concentrations of EB in formamide and real solution were calculated from standard curves of EB. Retinal EB leakage (ng) was equal to the actual concentration (in real solution) multiplied by 120 ml. The results were reported as ng/mg = standardized EB leakage (ng/mg)/retinal dry weight.

### Immunohistochemistry

Paraffin-embedded sections of retinas were dewaxed, diluted with distilled water, and blocked with 3% hydrogen peroxide for 15 min. Sections were then fixed in citrate under high pressure for 3 min, then incubated with primary antibodies against Claudin-5 (1:50 dilution) and Occludin (1:50 dilution) for 24 h at 4°C. The sections were washed with phosphate-buffered saline (PBS) three times and incubated with secondary antibodies (goat anti-rabbit, 1:200; goat anti-mouse, 1:200) for 2 h at the room temperature. Color Development Kits were used, with hematoxylin as a mild counterstain, and the sections were dehydrated in a graded series of ethanol concentrations and sealed. The mean optical densities of the sections were analyzed semiquantitatively with Image-Pro Plus analysis software (Media Cybernetics).

### Western Blotting

After 12 weeks of treatment, retinas of rats were harvested, 10 retinas were pooled as one sample for indicator detection by western blot. The retinas were washed with cold PBS, and incubated in RIPA buffer (Solarbio, China) on ice. Retinas were extracted, and protein content was quantified using a BCA assay kit (Cwbiotech, China). After loading buffer was added, cell lysates were separated by sodium dodecyl sulfate polyacrylamide gel electrophoresis on 12% gels and transferred to polyvinylidene fluoride membranes (Millipore, United States). Membranes were blocked using SuperBlock T20 (TBS) buffer, incubated with primary antibodies (Cell Signaling Technology, United States) overnight at 4°C, and washed three times with TBST. Dilution ratio: mouse monoclonal antibodies against VEGF (1:500), rabbit monoclonal antibodies against VEGFR (1:1000), mouse polyclonal antibodies against PEDF (1:500), rabbit polyclonal antibodies against Ang-1 (1:1000), rabbit polyclonal antibodies against Tie-2 (1:500), rabbit polyclonal antibodies against ZO-1 (1:500), rabbit monoclonal antibodies against Occludin (1:1000), mouse monoclonal antibody against Claudin-5 (1: 500). The membranes were then incubated with horseradish peroxidase-conjugated secondary antibodies (Jackson Immuno Research, United States) for 1 h, the dilution ratio: goat anti-rabbit, 1: 5,000; goat anti-mouse, 1: 5,000. Enhanced chemiluminescence agent (Millipore) was used to visualize bands of the antigen-antibody complexes, and an image analyzer (Bio-Rad, United States) was employed for densitometric analysis to obtain band intensities. Quantification of protein expression was normalized to glyceraldehyde 3-phosphate dehydrogenase (Abcam, United States).

### Real-Time Quantitative Reverse Transcription Polymerase Chain Reaction

Total RNA was extracted using a TRIzol reagant Kit (Gibco, NY, United States) according to the manufacturer’s instructions and stored at −40°C. Total RNA was reverse transcribed to first-strand cDNA using a Reverse Transcription Kit for 60 min at 42°C and 10 min at 70°C. cDNA was stored at −40°C until PCR. PCR was performed using a SYBR Green kit according to the manufacturer’s protocols. The primers sequences for ZO-1, Occludin, VEGF and reference gene sequence *β*-Actin used for amplification are shown in Table 1. DNA melting curves were generated, and relative quantities of gene expression were evaluated by PCR using the following three-step amplification procedure: one cycle at 95 °C for 10 min; 40 cycles at 95°C for 25 s, 55°C for 25 s, and 72°C for 50 s; and one cycle at 72°C for 8 min. The fluorescence signals were monitored, and three parallel tubes were prepared for each sample. Results were analyzed using the 2^-ΔΔCt^ method, and fold change relative to the control was determined.

**TABLE 1:** Primers sequences used for real-time PCR.

Genes	Forward primer	Reverse primer	Size (bp)
ZO-1	TCG​GAG​CTC​GGG​CAT​TAT​TC	CAG​GGC​ACC​ATA​CCA​ACC​AT	310
Occludin	CAA​CGG​CAA​AGT​GAA​TGG​CA	CTT​TCC​CCT​TCG​TGG​GAG​TC	182
β-Actin	CAC​CCG​CGA​GTA​CAA​CCT​TC	CCC​ATA​CCC​ACC​ATC​ACA​CC	207

### Quantitative Reverse Transcription Polymerase Chain Reaction for *miR-200b* Expression

We performed qRT-PCR for *miR-200b* expression with retinal samples. miRNA fractions were isolated from retinas using a miRNA isolation kit (CWbio, Co. Ltd.; cat. no. CW0627); U6-rRNA was used as an internal control. The primers sequences for miR-200b and U6-rRNA used for amplification are shown in Table 2. PCR was carried out using a miRNA Real-time PCR Assay Kit (CWbio Co. Ltd; cat. no. CW214). After the reaction, the Ct values of the samples were calculated at the point at which they reached the threshold value during the process of PCR amplification, and miR-200b levels were quantified based on the ratio of miRNA/U6-rRNA using these 2^-ΔΔ Ct^ method.

**TABLE 2:** Primers sequences used for real-time PCR.

Genes	Primer sequence
Rno-miR-200b	Forward primer: CAT​CTT​ACT​GGG​CAG​CAT​TGG
U6-forward	CTC​GCT​TCG​GCA​GCA​CAT​ATA​CT
U6-rerverse	ACG​CTT​CAC​GAA​TTT​GCG​TGT​C

**TABLE 3:** Composition of LTF.

Species	Herbal name	Place of origin	Part of use	Dosage (g)
*Astragalus mongholicus Bunge*	Astragali radix	Neimenggu	Root	45
*Salvia miltiorrhiza Bunge*	Salviae miltiorrhizae radix et rhizoma	Henan	Root and rhizoma	30
*Panax notoginseng (Burkill) F.H.Chen*	Notoginseng radix et rhizoma	Yunnan	Root	9
*Terminalia chebula Retz.; Terminalia chebula var. tomentella (Kurz) C.B.Clarke*	Hirudo	Shandong	Dry body	6
*Rheum officinale Baill.; Rheum palmatum L. Rheum tanguticum (Maxim.ex Regel)Balf*	Rhei radix et rhizoma	Gansu	Root and rhizoma	3

**TABLE 4:** Characterization of compounds in LTF.

Peak no	Compound	Formula	Content (mg/g)	Source
1	Calycosin-7-O-β-D-glucoside	C_22_H_22_O_10_	0.51	*Astragalus mongholicus Bunge*
2	Notoginsenoside R1	C_47_H_80_O_18_	0.45	*Panax notoginseng (Burkill) F.H.Chen*
3	Ginsenoside Rg1	C_42_H_72_O_14_	5.76	*Astragalus mongholicus Bunge; Panax notoginseng (Burkill) F.H.Chen; Salvia miltiorrhiza Bunge*
4	Salvianolic acid B	C_36_H_30_O_16_	7.03	*Salvia miltiorrhiza Bunge*
5	Ginsenoside Rb1	C_54_H_92_O_23_	0.54	*Astragalus mongholicus Bunge; Panax notoginseng (Burkill) F.H.Chen; Salvia miltiorrhiza Bunge*
6	Aloe-emodin	C_15_H_10_O_5_	0.024	*Rheum officinale Baill*
7	Rhein	C_15_H_8_O_6_	0.17	*Rheum officinale Baill*
8	Emodin	C_15_H_10_O_5_	0.0088	*Rheum officinale Baill*
9	Chrysophanol	C_15_H_10_O_4_	0.029	*Rheum officinale Baill*
10	Physcion	C_16_H_12_O_5_	0.11	*Rheum officinale Baill*
11	Tanshinone II	C_19_H_18_O_3_	0.0026	*Salvia miltiorrhiza Bunge*

### Statistical Analysis

Experiments were performed at least three times, and all values are expressed as means ± standard deviations. Data were evaluated using one-way analysis of variance (ANOVA), and post-hoc analysis with Bonferroni methods was used for multiple comparison. SPSS (version 20.0; IBM Corp., Armonk, NY, United States) was used for the analyses. Results with *p* values of less than 0.05 were considered statistically significant.

## Results

### Quality Control Analysis of Luo Tong Formula

**FIGURE 1:**
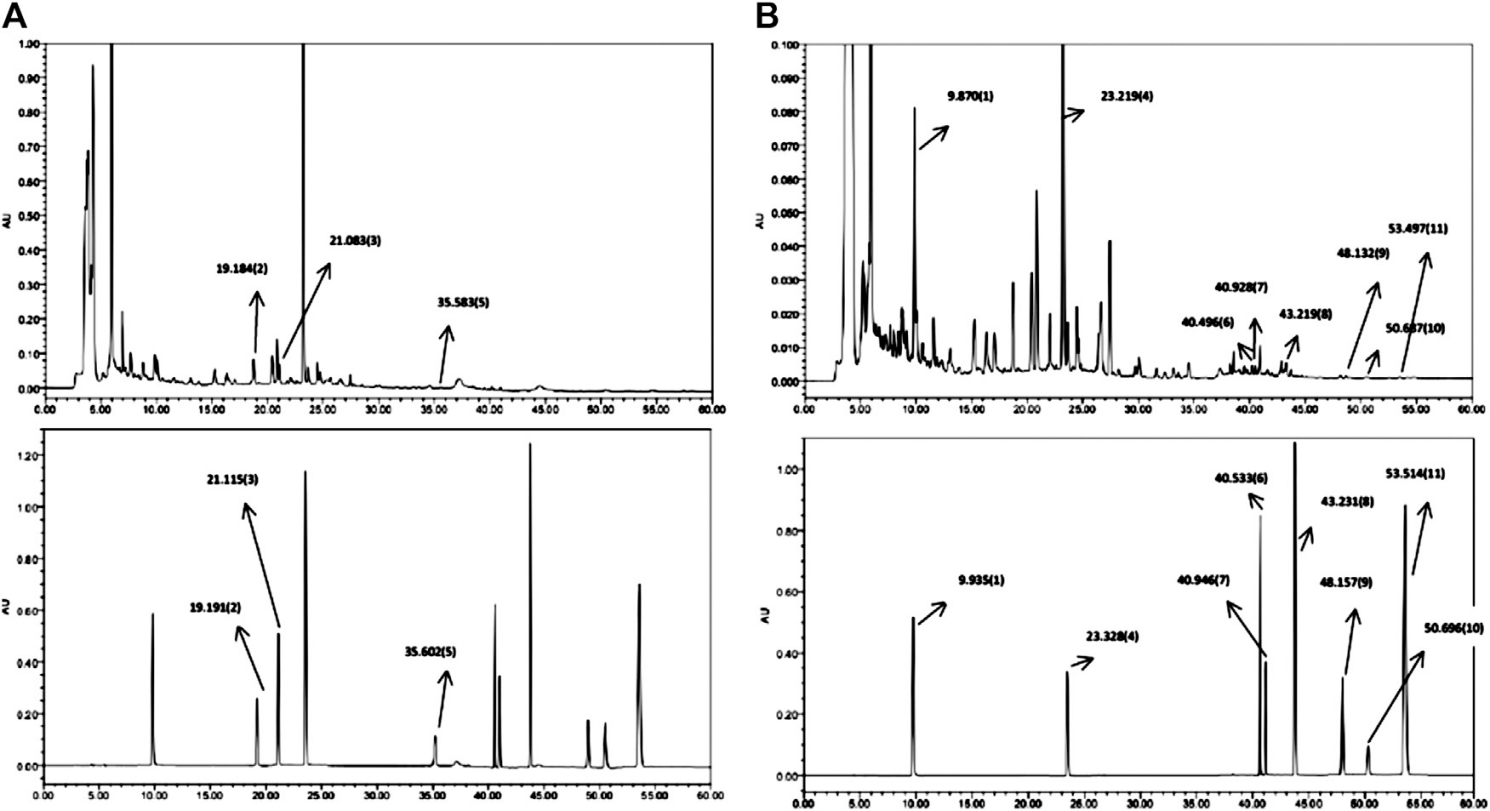
HPLC chromatogram of LTF. **(A)** Chromatogram at λmax = 210 nm. LTF is shown on top, while the reference standards are shown below. **(B)** Chromatogram at λmax = 254 nm. LTF is shown on top, while the reference standards are shown below.

To analyze and assess the quality of the LTF used in this study, chemical fingerprinting was performed using high performance liquid chromatography (HPLC). The species of the herbs found in LTF are listed in Table 3. Chromatograms of the five herbs of LTF were analyzed quantitatively at 210 nm (Figure 1A) and 254 nm (Figure 1B) using a standard curve, and the calculated concentrations (mg/g) of each compound are summarized in Table 4. The contents of Salvianolic acid B, Ginsenoside Rg1, Ginsenoside Rb1 and Calycosin-7-O-β-D-glucoside were greatest in LTF.

### Effects of Luo Tong Formula on Body Weight, Blood Glucose, and Hemorheology

**FIGURE 2:**
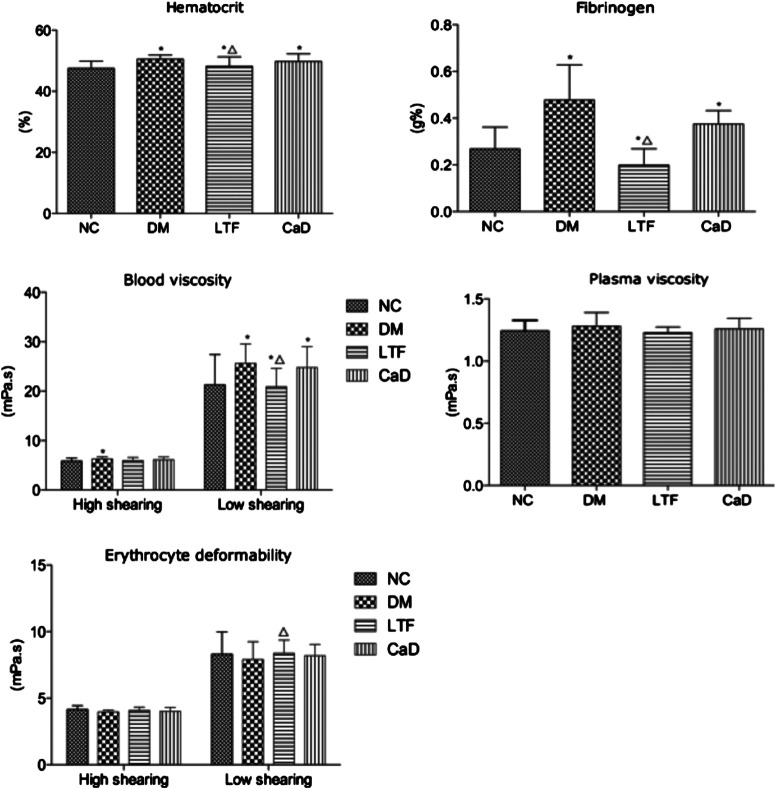
Effects of LTF and CaD on hemorrheological indicators. Data are mean ± SD from six rats per group. (*p < 0.05: vs. NC group; △ *p* < 0.05: vs. DM group).

Compared with the normal control (NC) group, diabetic group rats showed typical clinical features of DM, such as polydipsia, polyphagia, polyuria and weight loss, etc; they were also characterized by withered hair, cataract, loose stool. No rat sacrificed in the normal control group, three rats were sacrificed in the diabetic control group, three in the LTF group, and two in the CaD group at 12 weeks. Body weight and blood glucose were determined. Four weeks after STZ injection, body weights in the DM group were markedly declining compared with those in the normal control (NC) group. Body weights in the LTF and CaD groups were markedly elevated compared with those in the DM group. Additionally, fasting blood glucose levels in the DM group were markedly elevated compared with those in the NC group (*p* < 0.05). In contrast, fasting blood glucose levels in the LTF and CaD groups were not markedly declining compared with those in the DM group (*p* > 0.05) (Table 5). Hemorrheological indicators were also determined. Twelve weeks after STZ injection, hematocrit, blood viscosity (high shearing and low shearing), and fibrinogen levels were markedly elevated in the DM group compared with those in the NC group (hematocrit: 50.5 ± 1.38 vs. 47.5 ± 2.37; high shearing blood viscosity: 6.28 ± 0.43 vs. 5.85 ± 0.59; low shearing blood viscosity: 25.6 ± 3.95 vs. 21.3 ± 6.13; fibrinogen: 0.478 ± 0.15 vs. 0.268 ± 0.094; *p* < 0.05, respectively). The low shearing blood viscosity, hematocrit, and fibrinogen levels markedly improved after treatment with LTF (low shearing blood viscosity: 20.9 ± 3.73 vs. 25.6 ± 3.95; hematocrit: 48.2 ± 3.05 vs. 50.5 ± 1.38; fibrinogen: 0.199 ± 0.07 vs. 0.478 ± 0.15; *p* < 0.05, respectively), but no significant difference existed between the CaD and DM group (*p* > 0.05) (Figure 2).

**TABLE 5:** Changes in body weight and blood glucose for 0, and 12 Weeks.

Parameters	Weeks	Group			
		Normal	Diabetic	LTF	CaD
Weight, g	0	266.35 ± 13.47	267.38 ± 11.46	269.18 ± 12.81	264.93 ± 17.01
Blood glucose, mmol/L	12	512.80 ± 21.69	348.69 ± 23.66^*^	378.54 ± 25.47^*△^	375.62 ± 24.23^*△^
	0	5.21 ± 0.42	28.63 ± 5.48^*^	28.18 ± 5.53^*^	29.99 ± 4.14^*^
	12	5.23 ± 0.51	31.38 ± 2.20^*^	30.87 ± 2.62^*^	30.78 ± 2.82^*^

Note: Data are expressed as mean ± SD. N = 160 for 0 weeks, and n = 152 for 12 weeks. (*p < 0.05: vs. NC group; △ *p* < 0.05: vs. DM group).

### Effects of Luo Tong Formula on Histopathological Damage in the Retina

**FIGURE 3:**
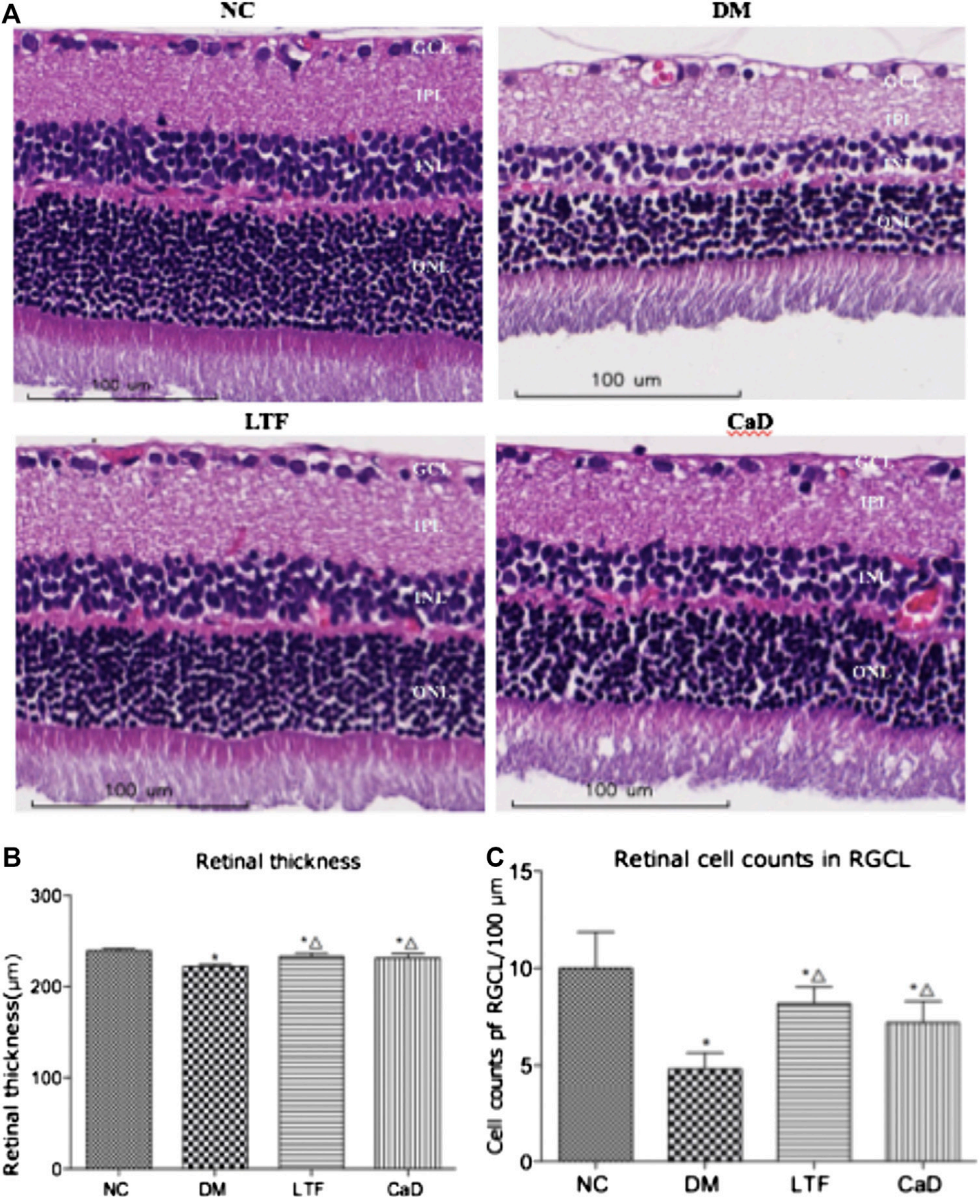
Effect of LTF and CaD on the retinal morphological changes. **(A)** HE staining of retina at 12 weeks (×400 magnification); **(B)** Toral retinal thickness; **(C)** Cell counts in the GCL; **(B–C)** Data are mean ± SD from six retinas per group. (*p < 0.05: vs. NC group; △ *p* < 0.05: vs. DM group).

Retinal sections followed by HE staining and retinal vascular digest preparations by PAS staining were determined after 12 weeks. The retinas of the rats in normal group presented a clear structure, and the cells in every layer were uniformly distributed and densely packed. However, the cells were irregularly organized and loosely arranged in the DM group. In addition, the retinal thickness and RGC number in the eyes of diabetic rats were decreased compared to those in the NC group (Retinal thickness: 222.2 ± 2.39 vs. 239.2 ± 2.68, *p* < 0.05; RGC number: 4.80 ± 0.84 vs. 10.00 ± 1.87, *p* < 0.05, respectively). Expectedly, LTF and CaD ameliorated the observed pathological changes, the retinal structure was improved, and the cells were organized with intact layers. Moreover, LTF and CaD increased the retinal thickness compared with those in the DM group (LTF: 233.2 ± 3.63 vs. 222.2 ± 2.39, *p* < 0.05; CaD: 231.4 ± 4.83 vs. 222.2 ± 2.39, *p* < 0.05, respectively), LTF and CaD also showed the increased number of RGC (LTF: 8.20 ± 0.84 vs. 4.80 ± 0.84, *p* < 0.05; CaD: 7.20 ± 1.10 vs. 4.80 ± 0.84, *p* < 0.05, respectively) (Figure 3).

**FIGURE 4:**
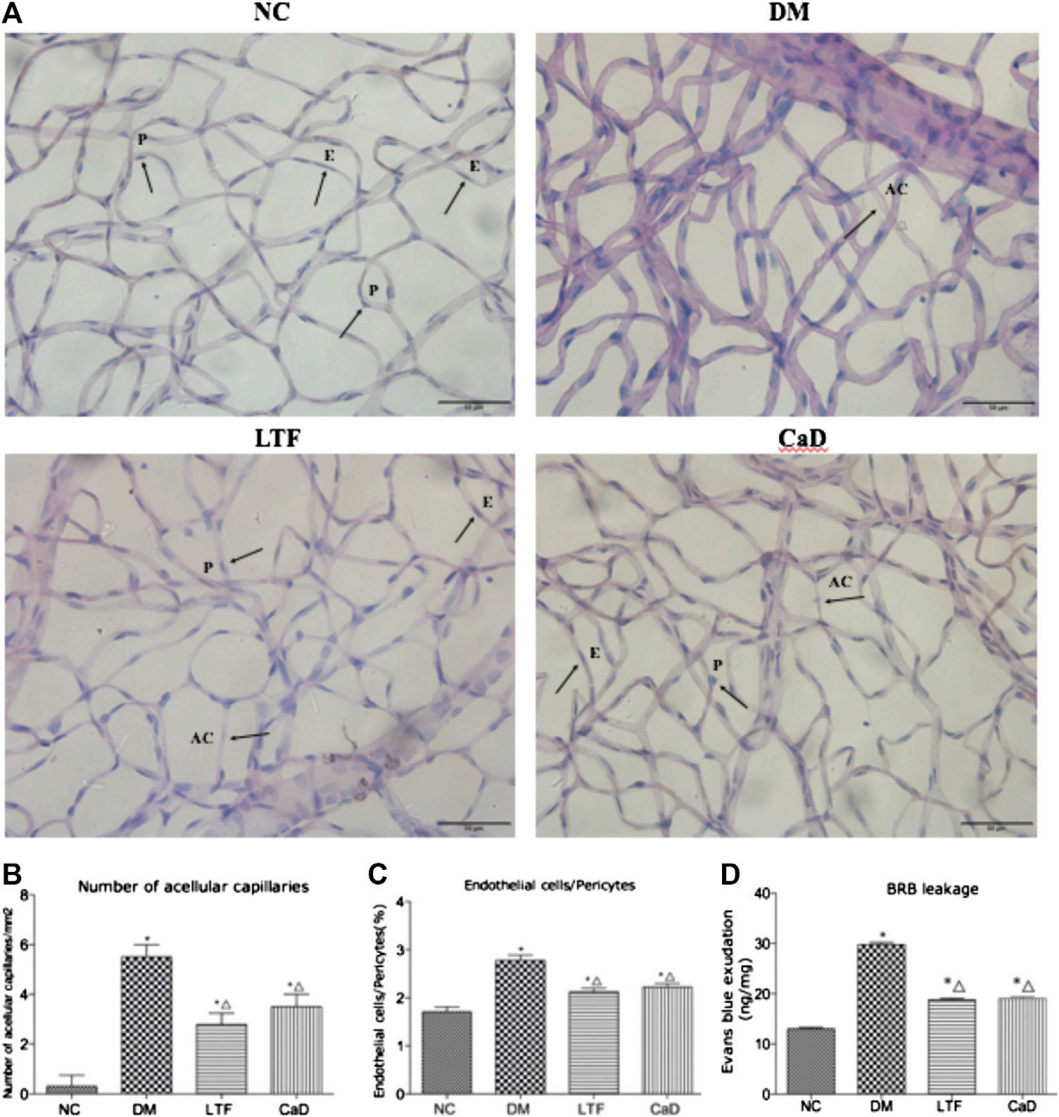
Effect of LTF and CaD on the retinal vascular morphological changes and BRB leakage. **(A)** PAS staining of retina at 12 weeks (400× magnification); **(B)** Number of acellular capillaries (Data are mean ± SD from six retinas per group); **(C)**The ratio of endothelial cells and pericytes (Data are mean ± SD from six retinas per group); **(D)** Measurement of BRB permeability (Data are mean ± SD from four retinas per group); (*p < 0.05: vs. NC group; △ *p* < 0.05: vs. DM group).

The nuclei of endothelial cells were oval, while the nuclei of pericytes were circular and the dye was darker in color compared to the endothelial cells. At 12 weeks, the ratio of endothelial cells to pericytes (E/P) were significantly increased in diabetic rats compared with that in the NC group (2.78 ± 0.11 vs. 1.71 ± 0.10, *p* < 0.05). LTF and CaD significantly moderate the ratio of E/P (LTF: 2.12 ± 0.08 vs. 2.78 ± 0.11, *p* < *0.05*; CaD: 2.22 ± 0.08 vs. 2.78 ± 0.11, *p* < *0.05*, respectively). Formation of acellular capillaries in the diabetic retinas was determined by PAS staining at 12 weeks. The results showed that acellular capillaries in DM group increased compared with normal control rats (3.57 ± 1.50 vs. 8.06 ± 1.32, *p < 0.05*). LTF and CaD significantly reduced acellular capillaries (LTF:5.45 ± 1.25 vs. 3.57 ± 1.50, *p* < 0.05; CaD: 5.29 ± 1.63 vs. 3.57 ± 1.50, *p* < 0.05, respectively). These findings suggest that a microvasculature lesion developed in the diabetic rat retinas and that LTF and CaD inhibited the pathological changes in DR (Figures 4A–C). Evans blue (EB) leakage was markedly elevated in the DM group compared with that in the NC group (29.77 ± 0.82 vs. 13.00 ± 0.57, *p* < 0.05). Additionally, EB leakage was markedly reduced in the LTF and CaD groups compared with those in the DM group (LTF: 18.77 ± 0.54 vs. 29.77 ± 0.82, *p* < 0.05; CaD: 19.02 ± 0.76 vs. 29.77 ± 0.82, *p* < 0.05, respectively) (Figure 4D).

### Effects of Luo Tong Formula on the Expression of Tight Junction Proteins in the Retina

**FIGURE 5:**
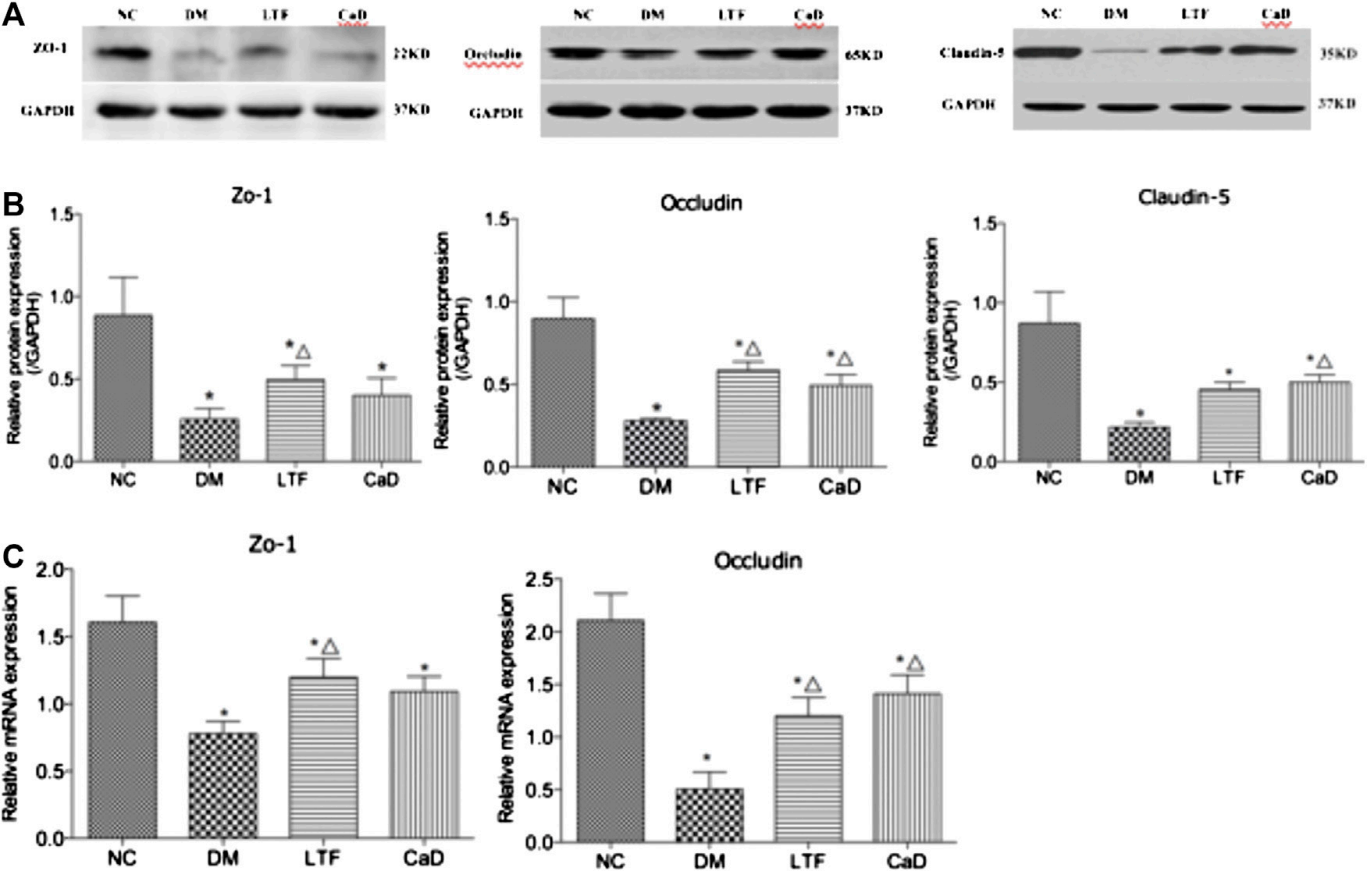
Effect of LTF and CaD on the ZO-1, Occludin and Claudin-5 expressions. **(A, B)** Western blot and quantitative measurement of retinal ZO-1, Occludin and Claudin-5; C Real-time PCR analysis of ZO-1 and Occludin. Data are mean ± SD from six rats per group, and the experiments were repeated independently four times (*p < 0.05: vs. NC group; △ *p* < 0.05: vs. DM group).

The expression levels of ZO-1, Occludin, and Claudin-5 in the retina were markedly down-regulated in the DM group compared with that in the NC group (ZO-1: 0.08 ± 0.08 vs. 0.89 ± 0.56, *p* < 0.05; Occludin: 0.28 ± 0.03 vs. 0.90 ± 0.30, *p* < 0.05; Claudin-5: 0.22 ± 0.08 vs. 0.87 ± 0.48, *p* < 0.05, respectively). Protein expression of ZO-1 was markedly up-regulated in the LTF group compared with that in the DM group (0.50 ± 0.21 vs. 0.08 ± 0.08, *p* < 0.05). Although ZO-1 protein expression in the CaD group was elevated, the difference was not significant (0.41 ± 0.25 vs. 0.08 ± 0.08, *p > 0.05*). Occludin protein expression was both markedly up-regulated in the LTF and CaD groups compared with that in the DM group (LTF: 0.59 ± 0.14 vs. 0.28 ± 0.03, *p* < 0.05; CaD: 0.49 ± 0.18 vs. 0.28 ± 0.03, *p* < 0.05, respectively). Claudin-5 protein expression was markedly up-regulated in the CaD group compared with that in the DM group (0.52 ± 0.12 vs. 0.22 ± 0.08, *p <* 0.05). However, although retinal expression of Claudin five was elevated in the LTF group, the difference was not significant (0.45 ± 0.12 vs. 0.22 ± 0.08, *p >* 0.05). (Figure 5).

### Luo Tong Formula Reversed the Expression of *miR-200b* in the Retina

**FIGURE 6:**
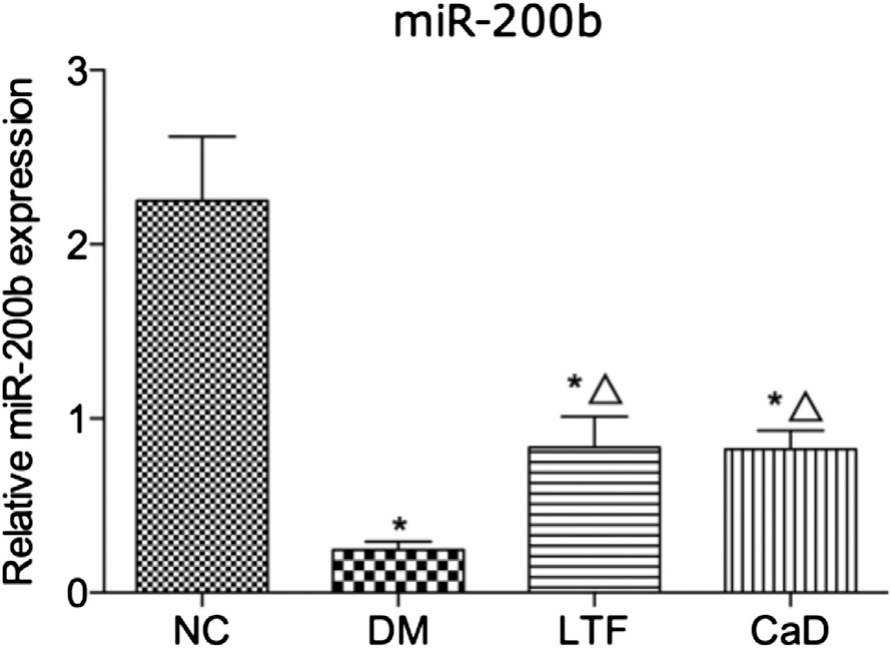
qRT-PCR of retinal miR-200b expression. Data are mean ± SD from six rats per group, and the experiments were repeated independently four times.(*p < 0.05: vs. NC group; △ *p* < 0.05: vs. DM group).

To determine the mechanisms through which LTF inhibited BRB leakage, we examined *miR-200b* expression. The expression of *miR-200b* in the retina was markedly down-regulated in the DM group compared with that in the NC group (0.24 ± 0.13 vs. 2.25 ± 0.90, *p* < 0.05). Additionally, retinal expression of *miR-200b* was markedly improved in the LTF and CaD groups compared with that in the DM group (LTF: 0.84 ± 0.43 vs. 0.24 ± 0.13, *p* < 0.05; CaD: 0.82 ± 0.26 vs. 0.24 ± 0.13, *p* < 0.05, respectively) (Figure 6).

### Luo Tong Formula Down-Regulated the Expression of the *miR-200b* Targets Vascular Endothelial Growth Factor, Vascular Endothelial Growth Factor Receptor, and Up-Regulated Pigment Epithelial-Derived Growth Factor in the Retina

**FIGURE 7:**
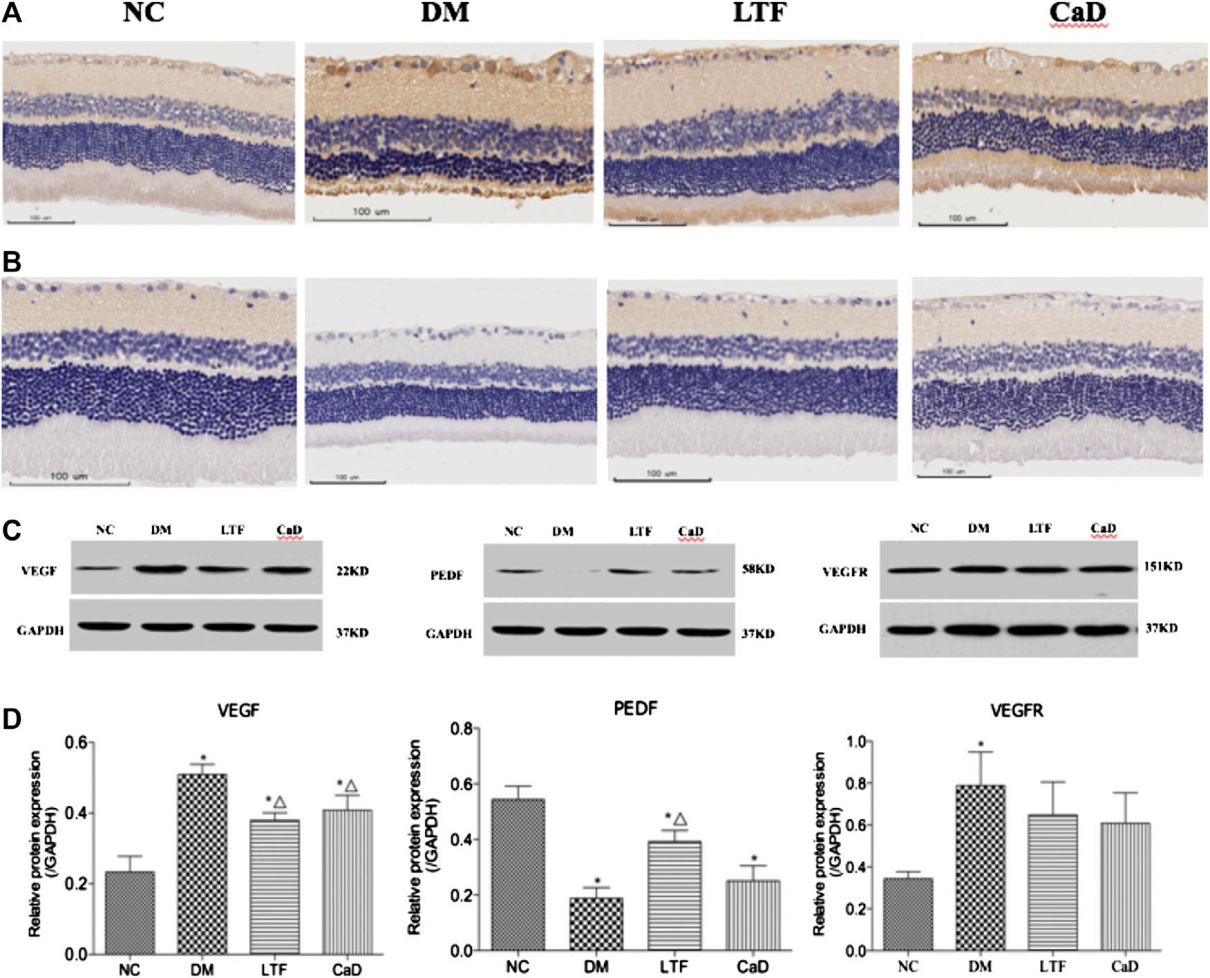
Effect of LTF and CaD on the VEGF, VEGFR and PEDF. **(A)** Immunostaining of retinal VEGF (400× magnification); **(B)** Immunostaining of retinal PEDF (400× magnification); **(C, D)** Western blot and quantitative measurement of retinal VEGF, PEDF and VEGFR. Data are mean ± SD from six rats per group, and the experiments were repeated independently four times. (*p < 0.05: vs. NC group; △ *p* < 0.05: vs. DM group).

VEGF and VEGFR protein expression levels were markedly up-regulated in the DM group compared with that in the NC group (VEGF: 0.51 ± 0.07 vs. 0.23 ± 0.11, *p* < 0.05; VEGFR: 0.79 ± 0.42 vs. 0.34 ± 0.08, *p* < 0.05, respectively). Additionally, VEGF expression was markedly down-regulated in the LTF and CaD groups compared with that in the DM group (LTF: 0.38 ± 0.05 vs. 0.51 ± 0.07, *p* < 0.05; CaD: 0.41 ± 0.11 vs. 0.51 ± 0.07, *p* < 0.05, respectively). In contrast, although VEGFR expression was down-regulated in the LTF and CaD groups, the difference was not significant (LTF: 0.65 ± 0.44 vs. 0.79 ± 0.42, *p* > 0.05; CaD: 0.59 ± 0.42 vs. 0.79 ± 0.42, *p* > 0.05, respectively). PEDF expression was markedly down-regulated in the DM group compared with that in the NC group (0.19 ± 0.11 vs. 0.54 ± 0.12, *p* < 0.05). Moreover, PEDF expression was markedly up-regulated in the LTF group compared with that in the DM group (0.39 ± 0.10 vs. 0.19 ± 0.11, *p* < 0.05). Although PEDF expression was up-regulated in the CaD group, the difference was not significant (0.25 ± 0.14 vs. 0.19 ± 0.11, *p* > 0.05) (Figure 7).

### Luo Tong Formula Up-Regulated Angiopoietin-1 and Tyrosine-Protein Kinase Receptor Expression in the Retina

**FIGURE 8:**
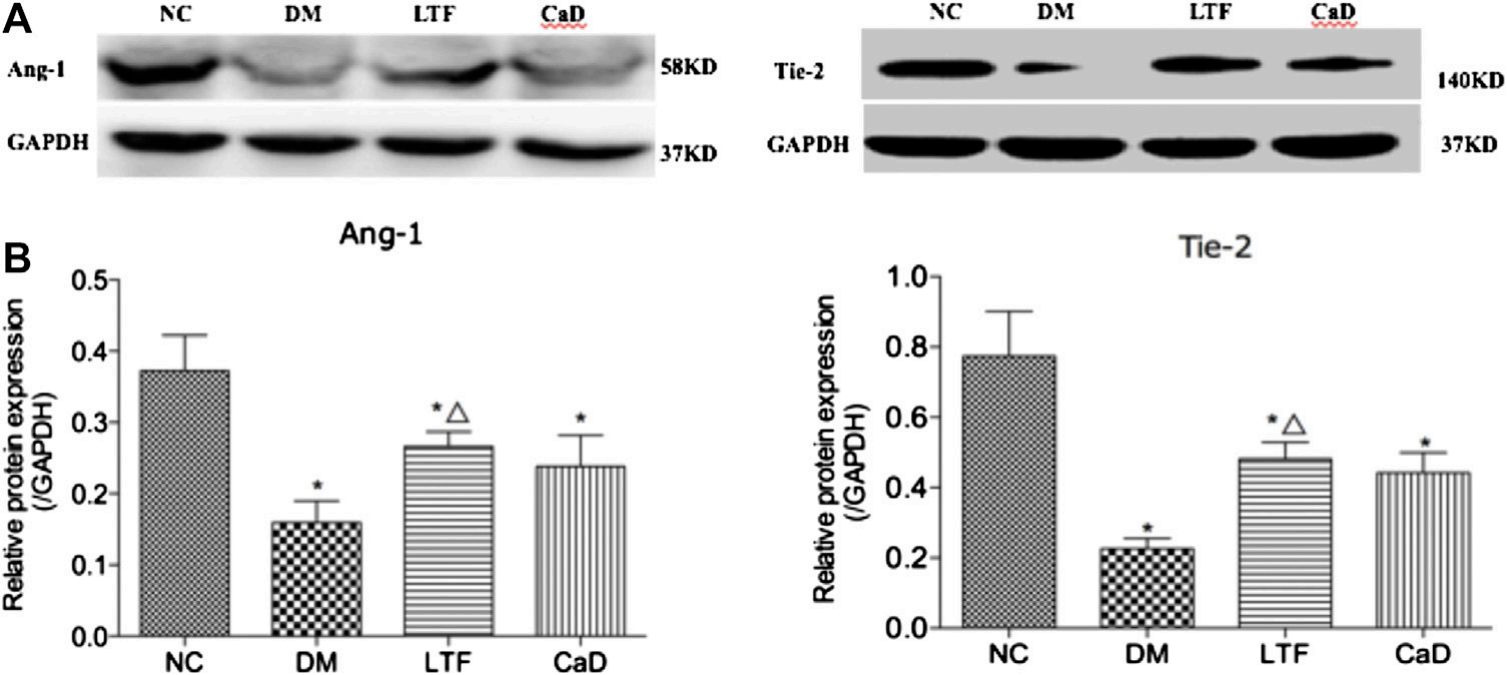
Effect of LTF and CaD on the Ang-1 and Tie-2. **(A, B)** Western blot and quantitative measurement of retinal Ang-1 and Tie-2. Data are mean ± SD from six rats per group, and the experiments were repeated independently four times (*p < 0.05: vs. NC group; △ *p* < 0.05: vs. DM group).

Retinal expression of Ang-1 and Tie-2 was markedly down-regulated in the DM group compared with that in the NC group (Ang-1: 0.16 ± 0.07 vs. 0.37 ± 0.12, *p* < 0.05; Tie-2: 0.23 ± 0.07 vs. 0.77 ± 0.34, *p* < 0.05, respectively). Additionally, retinal expression of Ang-1 and Tie-2 was markedly up-regulated in the LTF group compared with that in the DM group (Ang-1: 0.27 ± 0.05 vs. 0.16 ± 0.07, *p* < 0.05; Tie-2: 0.48 ± 0.13 vs. 0.23 ± 0.07, *p* < 0.05, respectively). Although retinal expression of Ang-1 and Tie-2 was up-regulated in the CaD group, the differences were not significant (Ang-1: 0.24 ± 0.11 vs. 0.16 ± 0.07, *p* > 0.05; Tie-2: 0.44 ± 0.15 vs. 0.23 ± 0.07, *p* > 0.05, respectively) (Figure 8).

## Discussion

**FIGURE 9:**
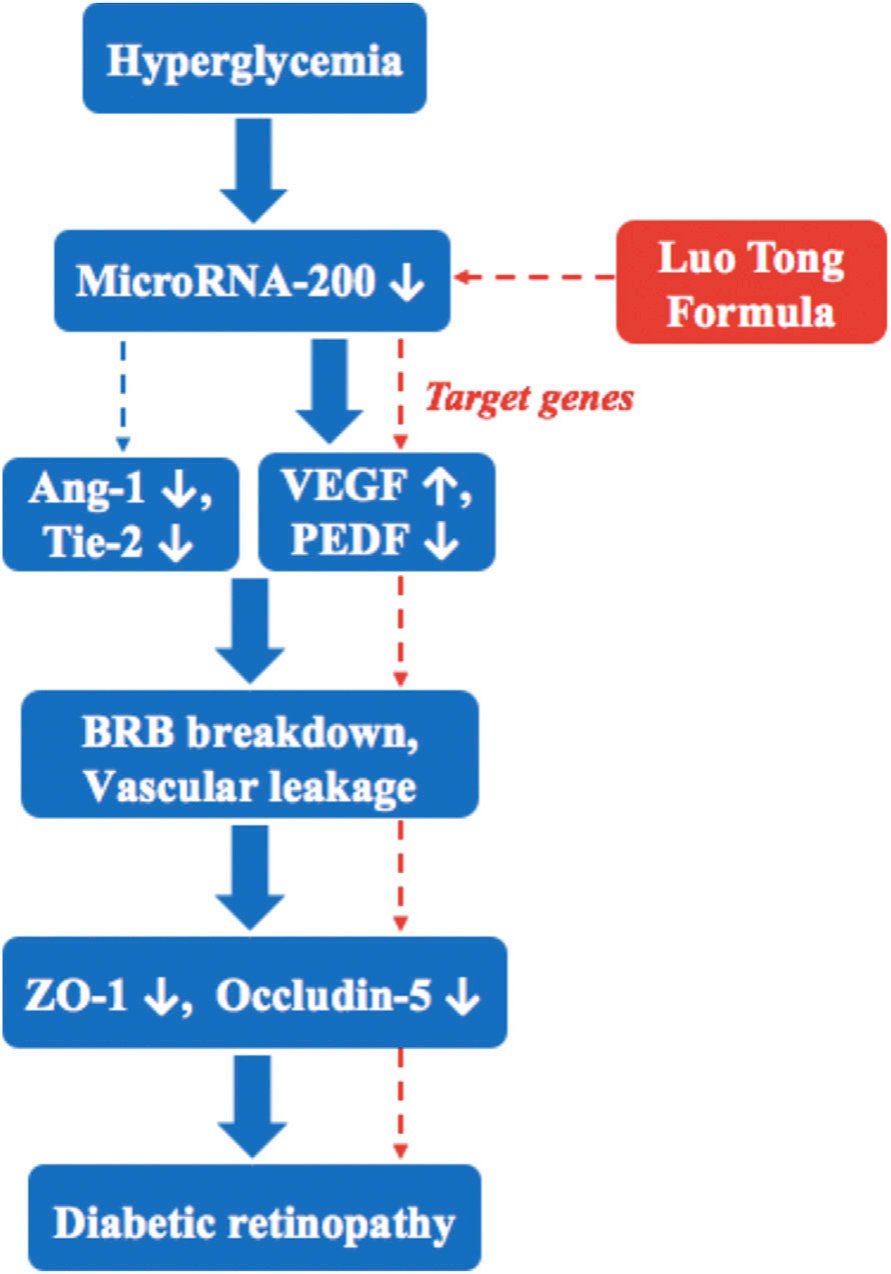
The miR-200b/VEGF pathway is involved in DR, which is inhibited by LTF.

LTF is a commonly used TCM prescription for treating diabetic microvascular complications (Zhou et al., 2000; Pang et al., 2020). In this study, we show that STZ-induced diabetes resulted in DR, and 12 weeks of LTF treatment promoted retinal microcirculation, attenuated vascular permeability, thereby ameliorating retinal damage and preventing DR. The mechanisms mediating these effects may be related to restoration of *miR-200b* expression, resulting in downregulation of the *miR-200b* targets VEGF and upregulation of PEDF, as well as increasing the expression of Ang-1 and Tie-2. This pathway is involved in DR, which is inhibited by LTF (Figure 9).

STZ-induced rats showed typical signs of retinal oxidative stress, retinal sorbitol pathway hyperactivity, poly (ADP-ribose) polymerase activation, caspases accumulation and NAD+/NADH redox imbalances during the early stages of DM, all of which represent a good model for DR (Obrosova et al., 2006; Jiang et al., 2015). Hyperglycemia induces endothelial dysfunction and retinopathy, which falls the category of “blood stasis” in TCM. Blood stasis is the core pathogenesis of DR (Pang et al., 2015; He et al., 2016; Ye et al., 2020). All herbal medicines in LTF had pro-microcirculatory properties and function by invigorating blood, dissolving stasis, and unblocking the collaterals according to the principles of TCM (He et al., 2016). In this study, we showed that LTF significantly decreased low shearing blood viscosity, hematocrit, and fibrinogen levels, thereby improving blood circulation. In order to highlight the feature of DR, we used 65 mg/kg STZ to establish the rat model, which made serious destruction of islet function and was similar to the pathogenesis of type 1 diabetes. Consequently, the blood glucose is difficult to control. Although the results indicated LTF had insignificant effect on lowering blood glucose, LTF inhibited the pathological changes in diabetic retinopathy and prevented capillary vasoregression through HE staining and PAS staining. In this study, we found that LTF reversed the ratio of E/P, thus inhibiting acellular capillary formation and vascular leakage. ZO-1, Occludin, and Claudin-5 are the main proteins involved in the tight junction complex, which is essential for endothelial cell integrity (Leal et al., 2010). Our results showed that LTF significantly reversed the down-regulation of ZO-1 and Occludin in the retina. Through this mechanism, LTF treatment may contribute to inhibiting BRB leakage. *miR-200b* is abundantly expressed in endothelial cells (McArthur et al., 2011; Ruiz et al., 2015). The expression of *miR-200b* under high-glucose conditions is still controversial. Several studies have suggested that *miR-200b* expression is significantly decreased in the diabetic rats and in human retinal vascular endothelial cells stimulated by hyperglycemia. Moreover, decreased *miR-200b* expression promotes the occurrence and progression of DR. However, Murray *et al.* (Murray et al., 2013) reported that *miR-200b* is upregulated in the retinas of Akita/+spontaneous diabetic mice, followed by downregulation of the oxidation resistance one gene and induction of oxidative stress in Müller cells. The disparities in miR-200b levels in the retina of diabetic models may be ascribed to different durations of diabetes or perhaps different models (genetic model of diabetes vs. STZ-induced diabetes). In this article, we found that retinal *miR-200b* expression was markedly down-regulated in DM rats compared with that in normal control group rats. However, LTF treatment significantly increased the expression of *miR-200b*. Notably, *miR-200b* targets VEGF, a key regulator of the development of DR, and downregulation of VEGF by *miR-200b* can lead to retinal microvascular leakage and BRB damage during all stages of DR (Ajlan et al., 2016). PEDF is required for the regulation of vascular permeability and angiogenesis. Studies have shown that decreased PEDF levels can further aggravate oxidative stress-induced apoptosis and pericyte loss, leading to vascular leakage, endothelial cell proliferation, and neovascularization (Zheng et al., 2009). More recent studies have suggested that reciprocal regulation between VEGF and PEDF may play an important role in retinal angiogenesis. PEDF significantly decreases VEGF expression in both retinal capillary endothelial cells (RCECs) and Müller cells, whereas VEGF markedly down-regulates PEDF expression in RCECs (Zhang et al., 2006; Zheng et al., 2009). Therefore, the balance between VEGF and PEDF may be important for DR. In this study, retinal VEGF and VEGFR expression in DM rats was markedly up-regulated compared with that in normal control group rats, whereas PEGF expression was markedly down-regulated. LTF treatment significantly decreased the expression of VEGF and increased the expression of PEDF. Thus, LTF treatment in DM rats improved vascular endothelial function by up-regulating *miR-200b* and subsequently decreasing VEGF expression and increasing PEDF expression. VEGFR is the receptor of VEGF, some components in LTF and CaD may combine with VEGF and act as an agonist, but can not directly affect on its receptors. Ang-1 is a growth factor that acts on vascular endothelial cells and specifically binds to Tie-2 on endothelial cells. Ang-1 inhibits BRB leakage and protects vascular endothelial cells from interleukin-mediated endothelial cell damage. Regulation of Ang-1 can help to prevent and treat DR (Joussen et al., 2002; Campochiaro and Peters, 2016). We found that LTF markedly up-regulated the expression of Ang-1 and Tie-2. However, further studies are still needed to determine whether the expression of Ang-1 and Tie-2 were affected by *miR-200b*.

There were several limitations to this study. First, the upstream mechanisms associated with the effects of LTF on *miR-200b* have not yet been clarified. Second, additional experiments in human retinal microvascular endothelial cells are needed to support the conclusion that LTF prevented DR. Third, the experiment failed to observe the effect of LTF on blood glucose and blood lipid due to type 1 diabetic rat model, and the new experimental animal models need to be applied.

## Conclusion

In summary, LTF treatment in a rat model of DR protected vascular endothelial cell function and attenuated vascular leakage, probably by restoring *miR-200b* expression, which resulted in downregulation of the VEGF/PEDF ratio and upregulation of Ang-1 and Tie-2.

## Data Availability Statement

All datasets presented in this study are included in the article/supplementary material.

## Ethics Statement

The animal study was reviewed and approved by the Ethics Committee of the Laboratory Animal Management of Guang’anmen Hospital of China, Academy of Chinese Medical Sciences.

## Author Contributions

XT, QN and BP conceived the study, and participated in its design together. SD and LD participate the animal administration and observation. BP, YQ and QL participated in immunohistochemistry work, Western blotting and RT-PCR study. ML and BP performed the statistical analysis. BP wrote the initial manuscript, QN helped to revise it critically, both of them are co-first authors. XT and ML are both corresponding authors. All authors read and approved the final manuscript.

## Funding

This study is supported by grants from National Natural Science Foundation of China (81774296); from Special program for excellent scientific personnel training of Chinese Academy of traditional Chinese Medicine (ZZ13-YQ-032); from Institutional Research Foundation of Guang’ anmen Hospital, China Academy of Chinese Medical Science (59957).

## Conflict of Interest

The authors declare that the research was conducted in the absence of any commercial or financial relationships that could be construed as a potential conflict of interest
