# Supplementary material for: Luo Tong Formula Alleviates Diabetic Retinopathy in Rats Through Micro-200b Target
Source: Front Pharmacol. 2020 Oct 30;11:551766. doi: 10.3389/fphar.2020.551766 (PMC7723456; doi:10.3389/fphar.2020.551766)
Supplement: Supplementary file 1 [file table1_v1.pdf]

Supplementary table

Herbal drug name	Full taxonomic names of all species
Astragali radix	Astragalus mongholicus Bunge
Salviae miltiorrhizae radix et rhizoma	Salvia miltiorrhiza Bunge
Notoginseng radix et rhizoma	Panax notoginseng (Burkill) F.H.Chen
Hirudo	Terminalia chebula Retz.; Terminalia chebula var. tomentella (Kurz) C.B.Clarke
Rhei radix et rhizoma	Rheum officinale Baill.; Rheum palmatum L. Rheum tanguticum (Maxim.ex Regel)Balf.
